# Clinical-applied anatomy of the carpal tunnel regarding mini-invasive carpal tunnel release

**DOI:** 10.1007/s00402-024-05560-7

**Published:** 2024-10-16

**Authors:** Peter Kaiser, Gernot Schmidle, Simone Bode, Ulrike Seeher, Hanne-Rose Honis, Bernhard Moriggl, Elisabeth Pechriggl, Hannes Stofferin, Marko Konschake

**Affiliations:** 1 Department of Orthopaedics and Traumatology, Medical University of Innsbruck, Innsbruck, Austria; 2 Department of Anatomy, Medical University of Innsbruck, Müllerstraße 59, Innsbruck, 6020 Austria

**Keywords:** Carpal tunnel, Median nerve, Berrettini, Release, Ultrasound, Surgery, Anatomy, Palmar arch

## Abstract

**Background:**

Carpal tunnel release is a widely performed procedure. Despite a high success rate, iatrogenic neurovascular injuries can occur which lead to a painful and unsatisfying outcome. This study conducted a detailed examination of the anatomy of the carpal tunnel and the proximity of neurovascular structures that are particularly susceptible to injury, especially in the context of minimally invasive carpal tunnel release procedures.

**Patients and methods:**

The anatomy of the carpal tunnel of 104 wrists of 52 body donors was examined. The precise anatomical location and the presence of variations were recorded for the median nerve, ulnar nerve, ulnar artery and Berrettini branch. The distance between the median nerve, the ulnar artery, the ulnar nerve, and the Berrettini branch was measured in a proximo-distal and radio-ulnar direction in relation to the distal ulnar end of the carpal tunnel.

**Results:**

The authors identified four main dangerous anatomical situations. (1) A proximal separation of the Long-Finger/Ring-Finger branch of the median nerve together with a narrow safe-zone; (2) an ulnar take-off of the recurrent muscle branch of the median nerve with a close radio-ulnar distance to the distal ulnar end of carpal tunnel; (3) an ulnar arterial arch lying close to the transverse carpal ligament; and (4) a proximal Berrettini branch also lying close to the latter. All situations are illustrated by photographs. Additionally, the authors present a sonographic carpal tunnel assessment protocol in order to reduce the risk of injury of any neurovascular structure in the proximity of the carpal tunnel.

**Conclusion:**

Certain patients may inherently face an increased risk of neurovascular injuries during minimally invasive carpal tunnel releases due to their anatomical variations. Four potentially risky scenarios were clearly illustrated. Consequently, one may consider conducting a preoperative ultrasound assessment of neurovascular structures at risk, when endoscopic or ultrasound-guided tunnel release are planned. In high-risk patients, open surgery should be preferred.

**Level of evidence:**

II.

## Introduction

The transverse carpal ligament has its attachments at the pisiform bone and the hook of the hamate on the ulnar side (Eminentia carpalis ulnaris), and at the scaphoid tubercle and the tubercle of the trapezium on the radial side (Eminentia carpalis radialis) [1]. Although most of its fibers run in a transverse orientation, they attach in a more circular fashion on the radial side and a more elongated manner on the ulnar side. The thickest portion of the ligament is located distally on the ulnar side and proximally on the radial side [2]. Biomechanically, the ligament stabilizes the bony structure of the carpus and acts as a pulley for the flexor tendons [3]. A compression of the median nerve is a well-known painful condition called carpal tunnel syndrome with an incidence of around 10.4/1000 person-years and a calculated prevalence of 10.6% independent of the study population [4].

In addition to non-surgical or conservative treatment, surgery can alleviate symptoms and pain by cutting or transecting this ligament. The procedure can be performed through an open incision, using endoscopic techniques, or with the guidance of ultrasound [5; 6]. Although carpal tunnel release shows a clinical success rate of 75–90% [7], surgical complications can occur in up to 5.6% and lead to unsatisfied patients [8].

Neurovascular structures that are particularly vulnerable to injury include the median nerve itself, the palmar cutaneous branch of the median nerve, the recurrent motor branch of the median nerve, the Berrettini branch, the nerve division for the third and fourth fingers, the superficial palmar arterial arch, as well as the ulnar artery and the ulnar nerve [9]. The aim of any surgical procedure should be a (nearly to) zero complication rate.

Therefore, the objective of this study was to conduct a detailed examination of the anatomy of the carpal tunnel and the proximity of neurovascular structures that are particularly susceptible to injury, especially in the context of minimally invasive carpal tunnel release procedures as the field of vision is either limited using an endoscopic approach or challenging in the inexperienced using ultrasound.

## Materials and methods

104 wrists of 52 whole bodies of body donors (mean 81 years, SD 9, 60–101 years; 25 males, 27 female) were examined. The human specimens were embalmed and preserved using an arterial injection of a formaldehyde/carbolsolution and immersion in phenolic acid in water for 1–3 months. The possibility of this solution causing preservation artefacts can be denied. The bodies were donated to the Institute of Clinical and Functional Anatomy of the Medical University of Innsbruck (MUI) by persons who prior to death had given informed consent for their use for scientific and educational purposes. Therefore, approval by an ethics committee is not necessary by Austrian law [10].

The wrists were dissected and released from the skin and palmar fascia including the palmaris longus tendon to visualize the carpal tunnel. Careful dissection of the neurovascular structures around the carpal tunnel was performed during the anatomical course by medical students and tutors. Measurements were conducted either by a specially trained anatomist, hand surgeon and intern after training. The proximal and distal width of the carpal tunnel was measured using two 1.0 mm K-wires, which were inserted along the path of the Flexor pollicis longus tendon radially and the flexor tendons next to the hook of the hamate ulnarly. The scaphoid, trapezium, hook of hamate and pisiform were marked using small flags. After measurement of the width of the carpal tunnel, a hook knife (Acufex ^®^, Smith & Nephew PLC, London, England) was inserted within the carpal tunnel ulnarly along the interdigitial path between the third and fourth finger, just next to the hook of the hamate inside the carpal tunnel. The hook was placed at the most distal and most ulnar end of the carpal tunnel. This point was defined as the main measurement point (P1) because it marks the optimal position of the hook knife and is highly reproducible. The distance between P1 and the distal end of the hook of the hamate was measured in order to define the exceeding distance of the transverse carpal ligament distal to the hook.

**Fig. 1:**
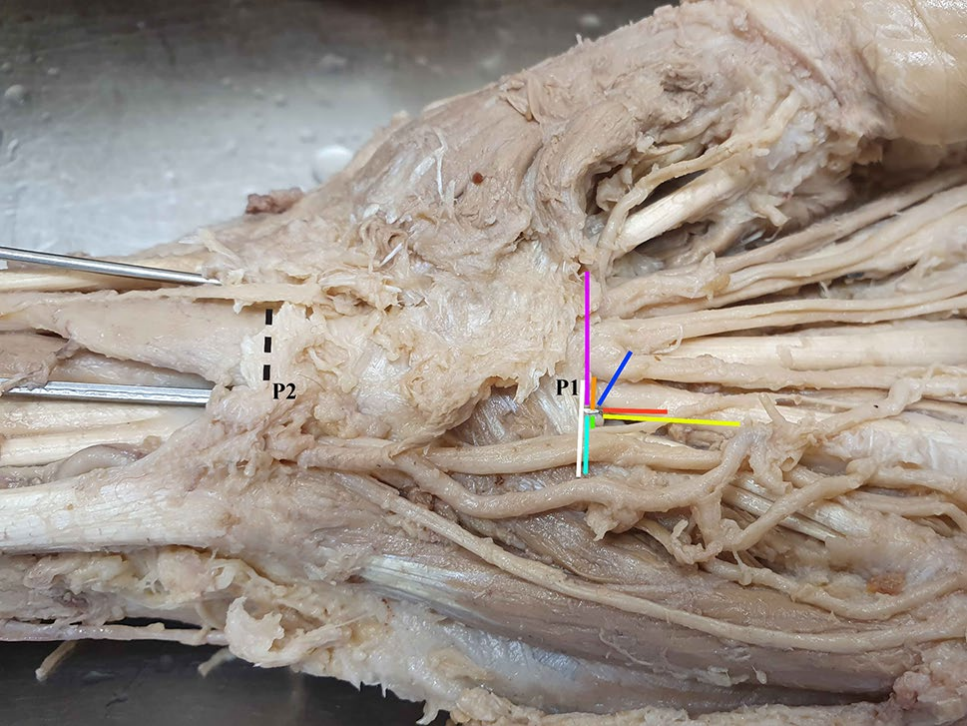
Measurements referenced to P1 and P2 (White: Distance between UA and MN; Turquoise: Distance between P1 and UA radio-ulnarly; Purple: Distance between P1 and RBMN; Orange: Distance between P1 and MN; Blue: Distance between P1 and separation of Median Nerve N3/4; Red: Distance between P1 and a Berrettini branch; Yellow: Distance between P1 and UA proximo-distally; Black: Distance between P2 and the PCBMN

The median nerve (MN) was explored for normal singular anatomy or for a bifid nerve and a median artery. The recurrent motor branch of the median nerve (RBMN) was dissected and classified according to the Lanz classification [11; 12]. The radio-ulnar distance between the median nerve as well as its motor branch and P1 were measured. The palmar cutaneous branch of the median nerve (PBMN) was visualized and explored whether it showed a radial or ulnar take-off and if it pierced the transverse carpal ligament. The radio-ulnar distance between this branch and the hook knife was measured at the proximal ulnar boarder of the transverse carpal ligament (measurement point P2). This point was used because the PBMN does not reach as far distal as P1 and can travel proximally ulnar to the MN in very rare cases. The separation of the third common branch of the median nerve (long finger-ring finger branch – LFRF) was explored whether it separated proximally or distally of the distal end of the carpal tunnel and the distance of this nerve to P1 was measured. The presence of the Berrettini branch (BB – a sensory ulnar-to-median nerve connection) was noted including a classification according to Ferrari and Gilbert [13; 14], measurement of the distance to P1, measurement of the seperation angle and anatomical position regarding the ulnar nerve and artery. The presence and type of the superficial palmar arterial arch (SPA) was classified according to Srimani and Saha [15] and its proximo-distal distance to P1 measured. The distance between P1 and the ulnar artery as well as ulnar nerve were measured in a radio-ulnar direction. The position of the ulnar artery (UA) and ulnar nerve (UN) regarding the hook of the hamate were also noted. The safe-zone between the ulnar artery and median nerve was measured. At last, the transverse carpal ligament was transected and its length measured. Nakamichi et Tachibana do not recommend an ultrasound guided carpal tunnel release in case of a safe zone of 3 mm or below [16]. Therefore, the frequency of cases with a safe zone or a distance of P1 to neurovascular structures of below or equal 3 mm and 5 mm was recorded. Because of different sizes of hook knives or needles and the need for an adequate and safe working distance, the authors also reported on the 5 mm distance between P1 and neurovascular structures. Measurements exceeding this limit were classified as “dangerous” for a minimally invasive release. All distances measured to the reference point P1 are illustrated in Fig. 1. Deduced from the findings of the present study, a diagnostic ultrasound evaluation workflow was developed for clinical practice that may display specific anatomic hazards for carpal tunnel surgery.

Data were reported using descriptive statistics calculated by SPSS Statistics (version 23, IBM, Armonk, NY, USA).

## Results

The mean length of the TCL (transverse carpal ligament) was 25 mm (SD 3 mm; range 17–32 mm), the proximal width 17 mm (SD 3 mm; range 9–25 mm) and the distal width 15 mm (SD 3 mm; range 9–24 mm).The proximo-distal distance between the distal end of the hook of hamate to P1 was 5 mm (SD 2 mm; range 2–12 mm).

A bifid MN (median nerve) was found in 13 cases. A median artery was present in 10 cases - one case with a single MN and 9 cases with a bifid MN. The division of the bifid MN was 19 mm (SD 19; range 2–56) proximal to the proximal end of the TCL. None of the MN branches were in an own compartment within the carpal tunnel. The radio-ulnar distance between the MN and P1 was 6 mm (SD 2; range 1–13).

**Fig. 2:**
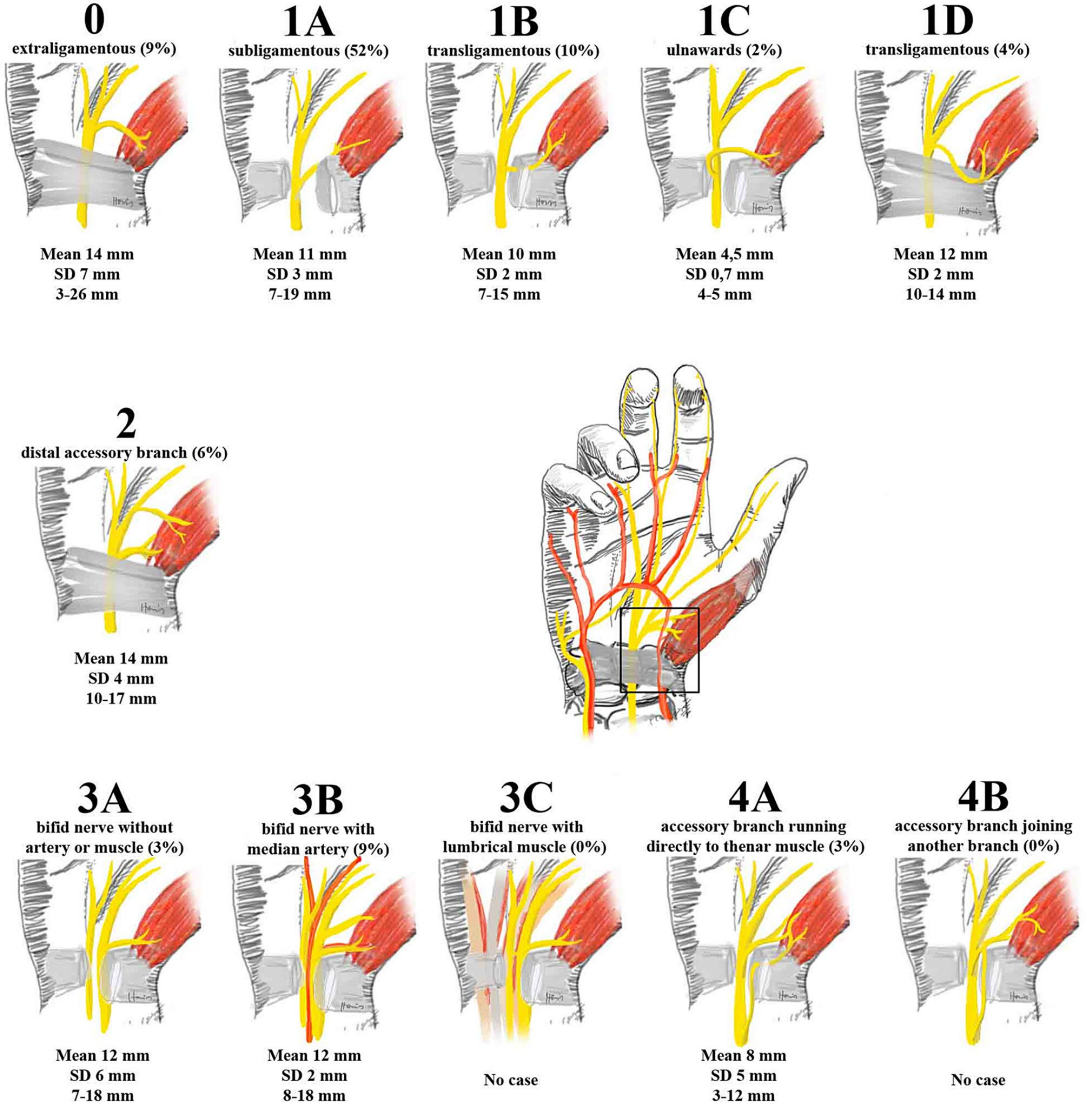
Distribution of the RBMN according to the Lanz classification including the mean distance (standard deviation and range) of P1 to RBMN

Figure 2 shows the distribution of the Lanz classification of the RBMN (recurrent motor branch of the median nerve) and the radio-ulnar distance between P1 and the RBMN for each type. The mean distance between P1 and the RBMN was 12 mm (SD 4 mm, range 3–26 mm).

The PBMN (palmar cutaneous branch of the median nerve) separated from the MN 46 mm (SD 20 mm; range 11–105 mm) proximal to the proximal end of the TCL. It showed an ulnar-take off in only one case while all others had a radial take-off. The radio-ulnar distance between the PBMN and P2 was 16 mm (SD 6; range 5–38 mm).

The common LFRF (long finger-ring finger branch) separation was proximal to the TCL in 37 cases (3 mm proximal to distal end of TCL excluding all bifid MN [SD2 mm; range 1–9 mm]), distal in 53 cases (4 mm distal to distal end of TCL excluding all bifid MN [SD 2 mm; 1–10 mm]) and directly at the end of the TCL in 14 cases (0 mm distance to distal end of TCL). Only 3 cases showed a separation distal to the SPA (superficial palmar arterial arch). The radio-ulnar distance between the LFRF separation and P1 was 8 mm (SD 7 mm; 2–71 mm.

**Fig. 3:**
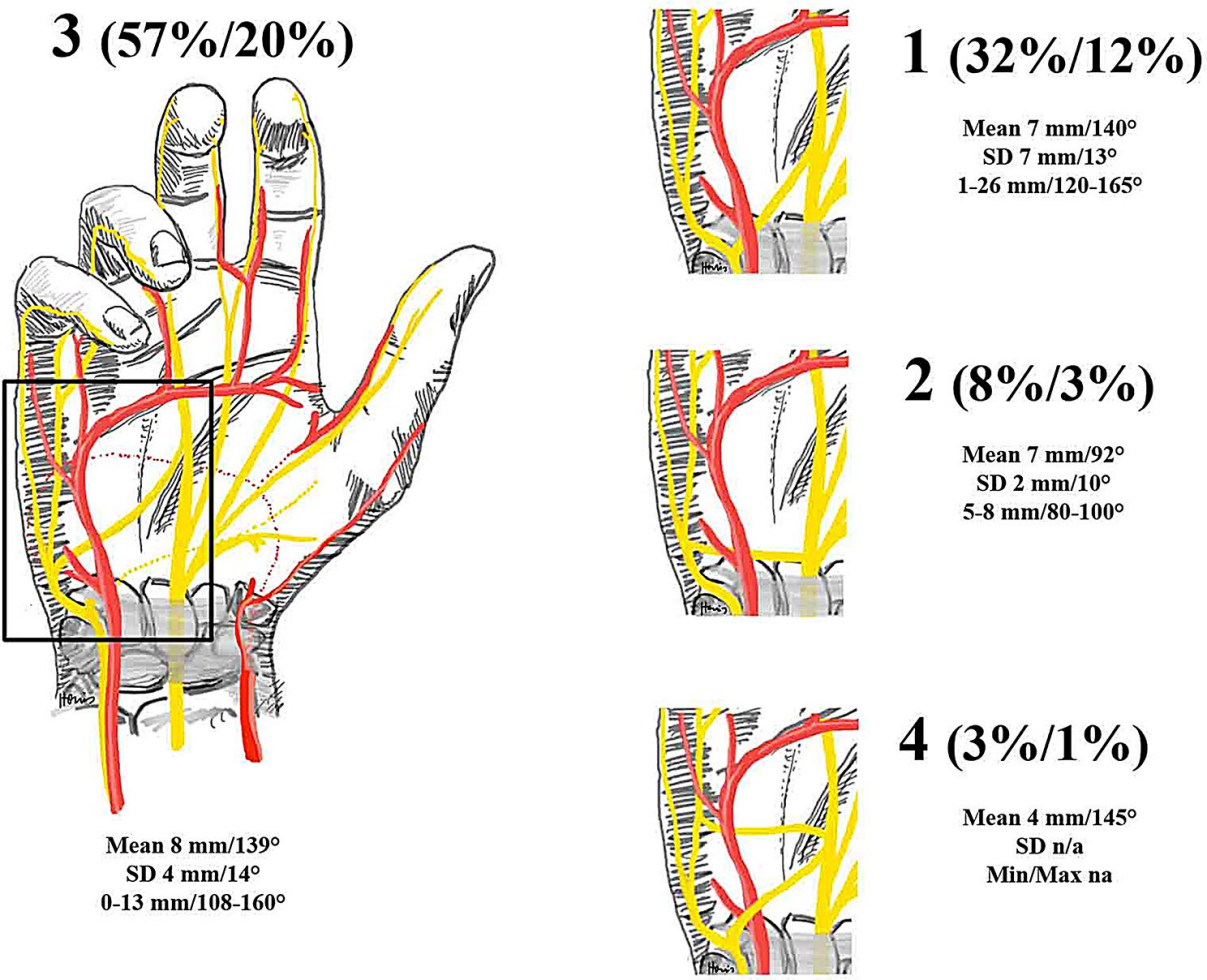
Distribution of the BB (in relation to 37 cases with a BB and in relation to all 104 hands) including the mean distance (standard deviation and range) and separation angle of P1 to BB

The BB (Berrittini branch) was found in 37 cases (36%). Figure 3 shows the distribution according to the Ferrari and Gilbert classification [13; 14], the proximo-distal distance between the BB and P1 and the separation angle of the BB. The mean proximo-distal distance between the BB and P1 was 7 mm (SD 5 mm, range 0–26 mm). 31 cases separated proximal to the SPA, 5 distal and one had a proximal and distal separating branch. All BB were dorsal to the ulnar artery except one that was palmar to the artery and one that showed a palmarly and dorsally situated BB.

Figure 3: Distribution of the BB (in relation to 37 cases with a BB and in relation to all 104 hands) including the mean distance (standard deviation and range) and separation angle of P1 to BB.

**Fig. 4:**
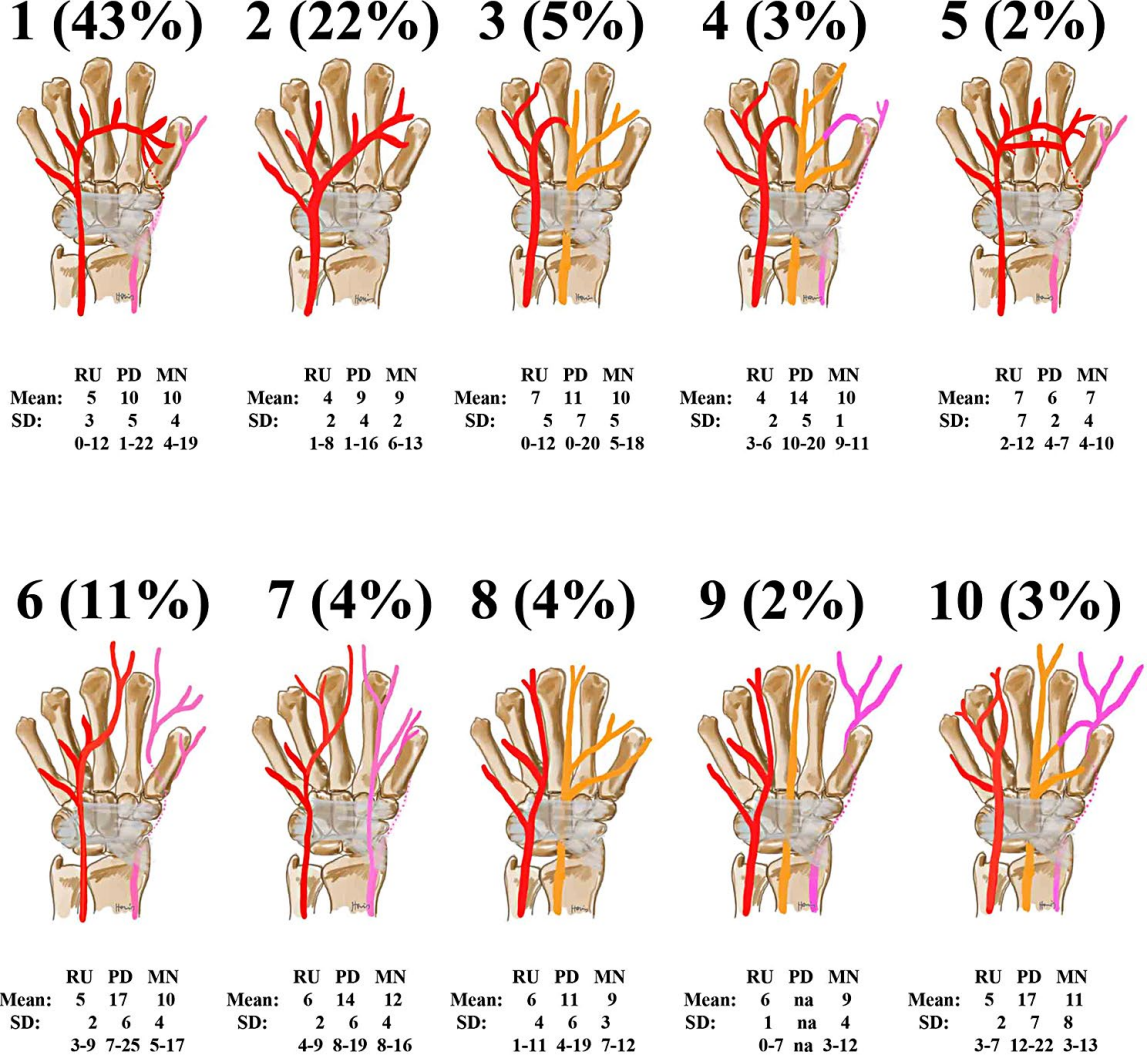
Distribution of the UA including the mean distance (standard deviation and range in mm); RU – radioulnar distance between P1 and UA; PD – proximo-distal distance between P1 and UA; MN – radioulnar distance between UA and MN

Figure 4 shows the distribution of the types of the SPA and the radio-ulnar distance between the UA (ulnar artery) and P1, the proximo-distal distance between the SPA and P1 and the radio-ulnar distance between the UA and the MN. The mean proximo-distal distance between P1 and the artery was 11 mm (SD 6 mm; range 0–25 mm) and radio-ulnar was 5 mm (SD 3 mm; range 0–12 mm). The mean radio-ulnar distance between the MN and the UA was 10 mm (SD 3 mm, range 4–19 mm).

34 UA were situated ulnarly (2 mm; SD 1 mm, range 1–6 mm) to the hook of hamate, 68 centrally on the hook of hamate (0 mm) and one artery was lying radially to the hook of hamate (1 mm).

The UN was situated ulnarly to the hook of hamate in 92 cases (2 mm; SD 2 mm, range 1–8 mm) and centrally over the hook in 12 cases (0 mm).The radio-ulnar distance between the UN and P1 was 8 mm (SD 3 mm, range 3–14 mm).

Case counts of distances ≤ 3 mm, < 5 mm and ≤ 5 mm between MN, RBMN, LFRF, BB, SPA, MN and UN and P1 are shown in Table 1. 27 cases (26%) were classified as dangerous because of a distance to any neurovascular structure below or equal 3 mm, 57 cases (55%) below 5 mm and 73 cases (70%) below or equal 5 mm.

**Fig. 5:**
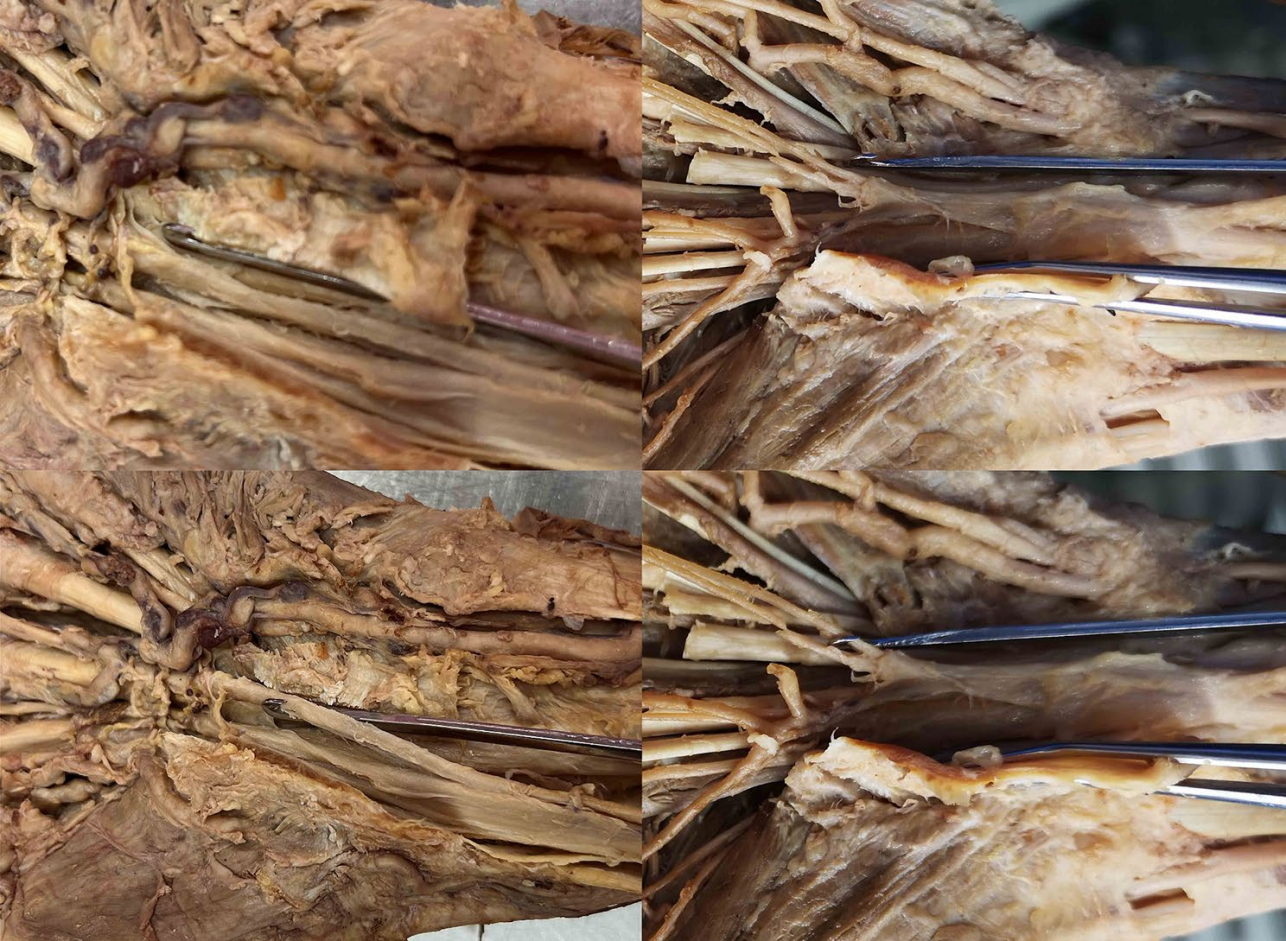
The hook knife is situated ulnarly to the median nerve (upper two images). In cases of a proximal separation of the common long finger-ring finger branch, the hook knife may be dangerously hooked around this branch and injure it (bottom left). In cases of a proximal separation of both branches of the common long finger-ring finger branch, the hook knife may be dangerously hooked around one of the branches and injure it (bottom right)

**Fig. 6:**
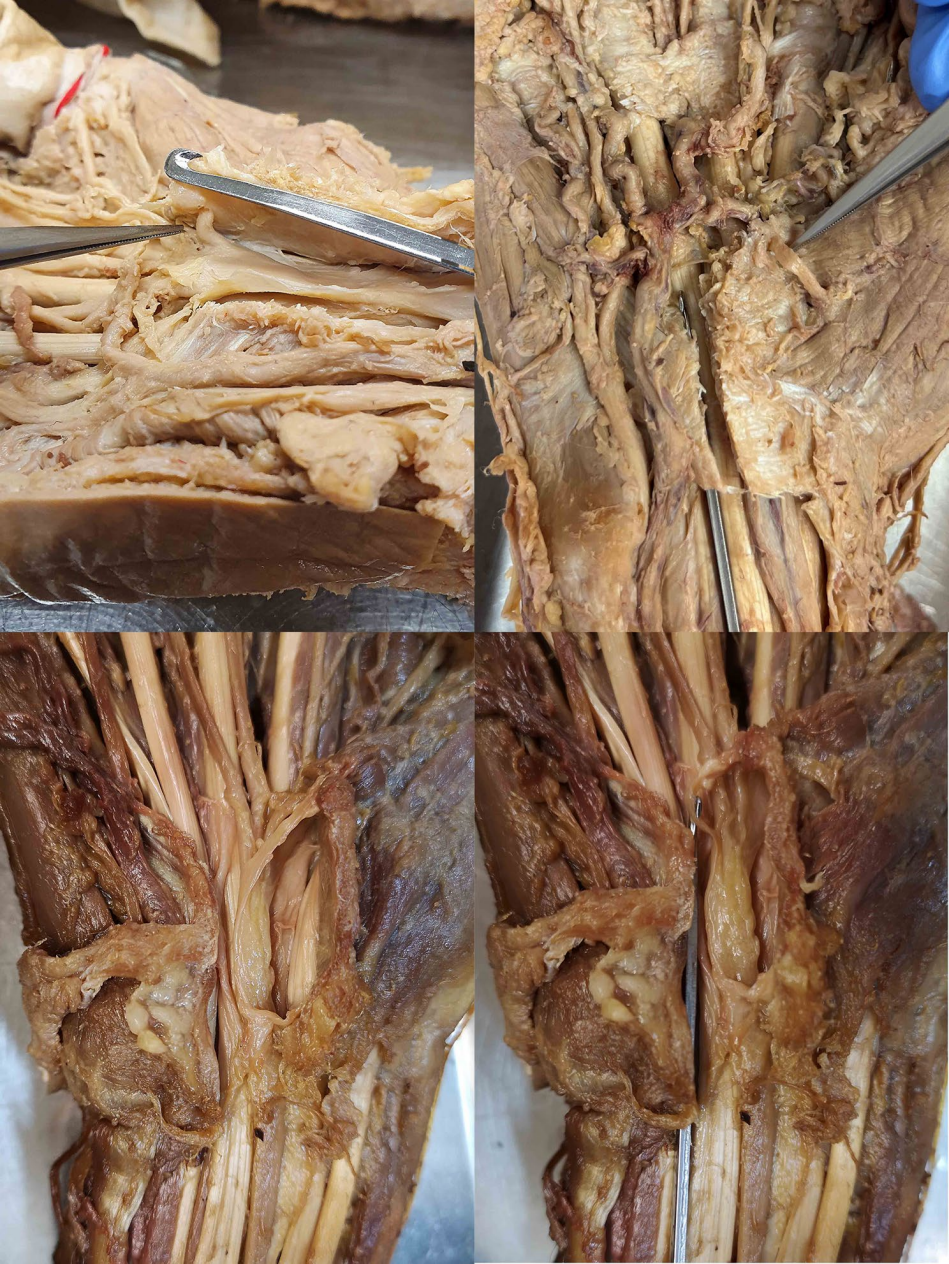
The RBMN is usually situated far away of the hook knife radially in a Lanz type 1B (upper left) and 1 A (upper right). In cases of a type 1 C the ulnarly positioned nerve is in high danger because the hook knife can hook around the nerve in its ulnar trajectory (bottom two images)

**Fig. 7:**
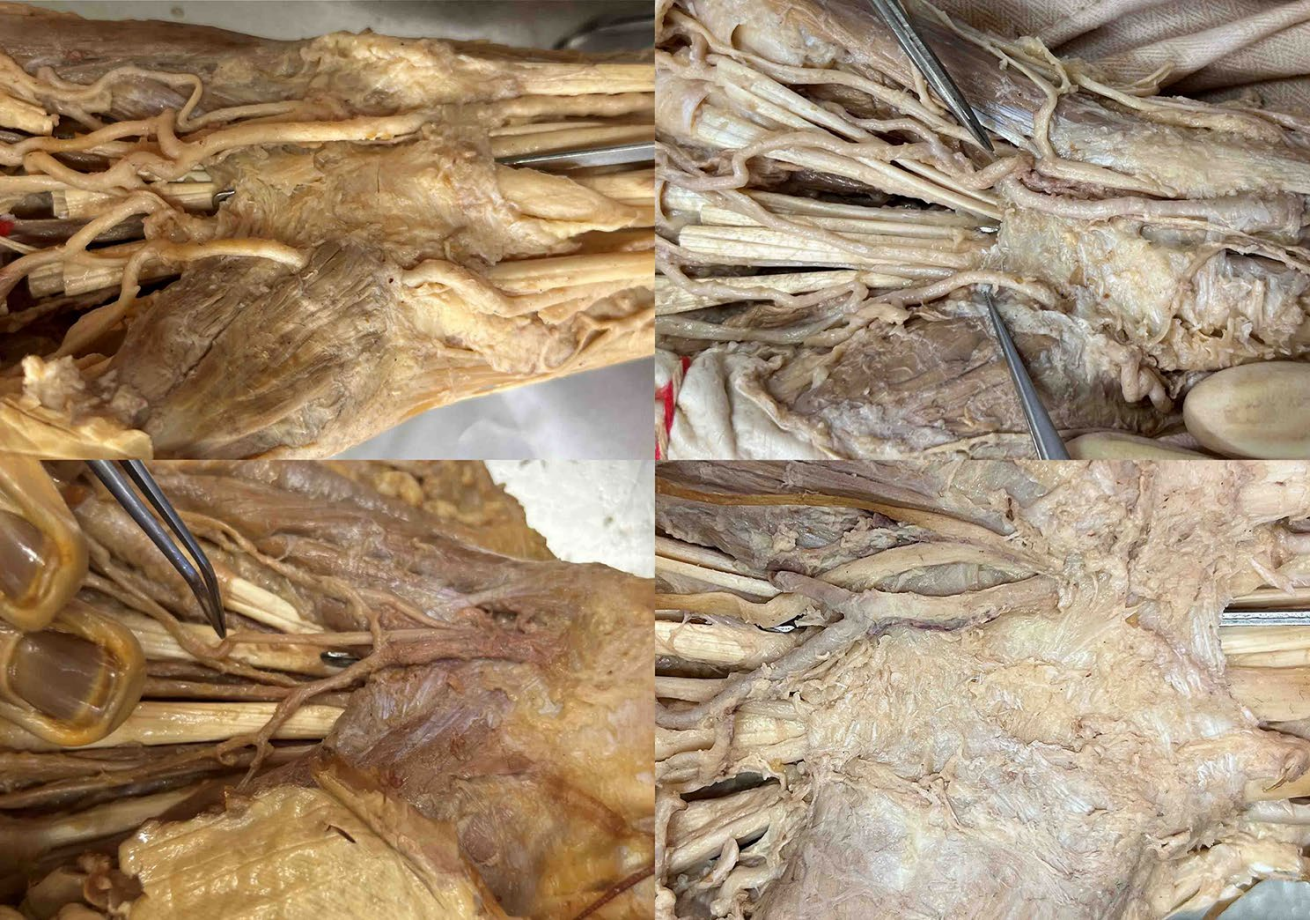
The ulnar artery is typically situated far distally from the distal end of the carpal tunnel, so there is minimal danger of injuring this vessel or the arch (upper left). The superficial arch can also be absent leading to no danger of injury (upper right). However, the artery or arch can be situated radially to the hook of hamate or turn just on the distal end of the carpal tunnel with no distance to the hook knife. Thus, the knife can hook around the vessel and injure it (bottom both images)

**Fig. 8:**
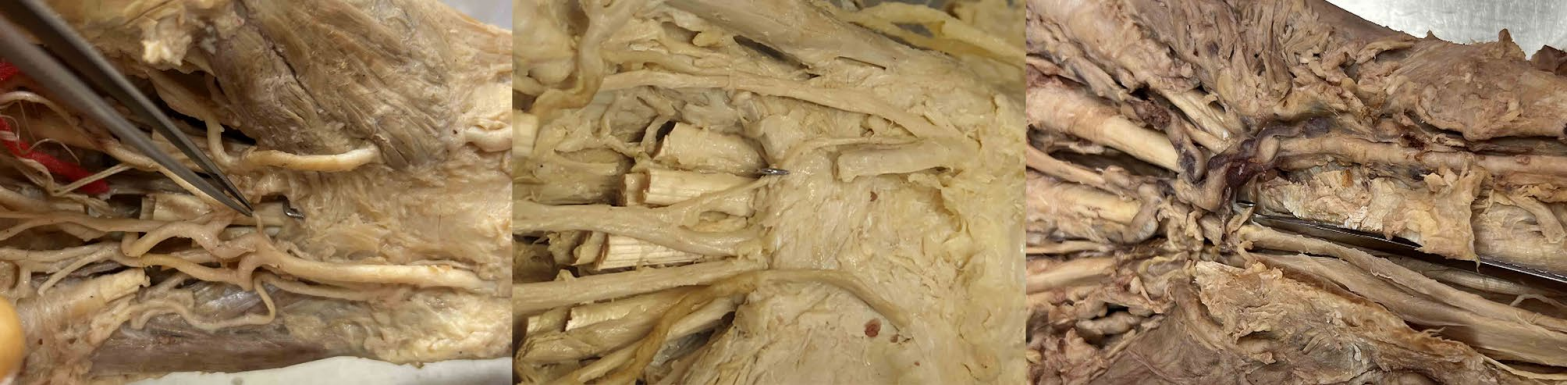
The presence of a Berrettini branch is highly variable. If present, the most cases show a distal position, far away from the end of the carpal tunnel and the hook knife (Type 3, left image). However, a type 1 (middle image) and type 2 (right image) Berrettini branch with a close situation on or near the distal end of the carpal tunnel can be highly dangerous, as the hook knife may potentially hook around this nerve

The authors identified 4 main dangerous situations (Figs. 5, 6, 7 and 8):

1. a proximal separation of the LFRF with a narrow safe-zone.

2. ulnar take-off of the RBMN with a close radio-ulnar distance to P1.

3. an ulnar artery/arterial arch lying close to or on the distal end of the TCL.

4. a proximal Berrettini branch (Type 1 or Type 2) lying close to the distal end of the TCL.

**Fig. 9:**
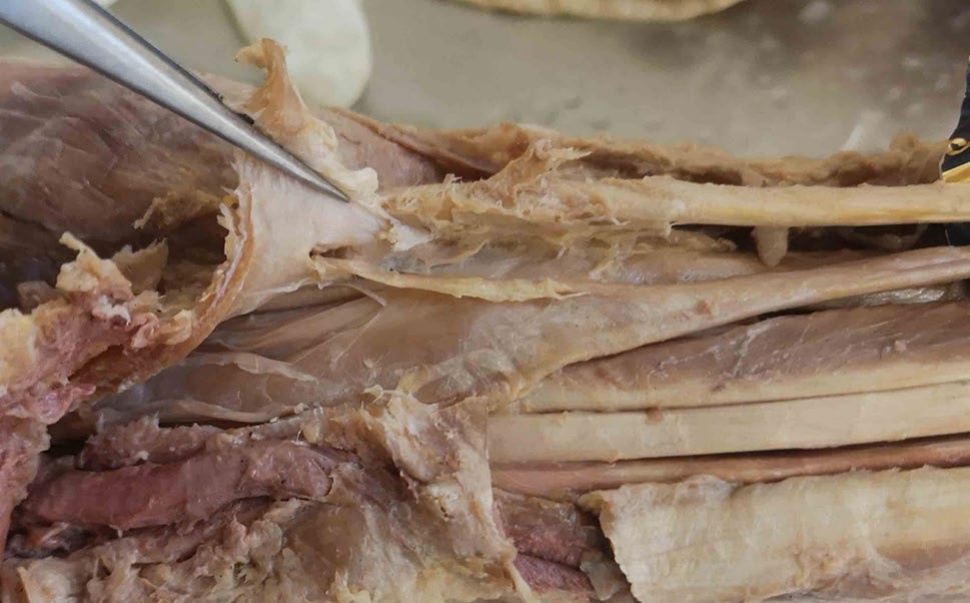
The PBMN was always situated far radially away from the trajectory of the hook knife and never in danger – even in cases with a transligamentous pathway of the PBMN

The PBMN was not at danger in any of our cases due to the ulnar trajectory of the hook knife (Fig. 9).

**Fig. 10:**
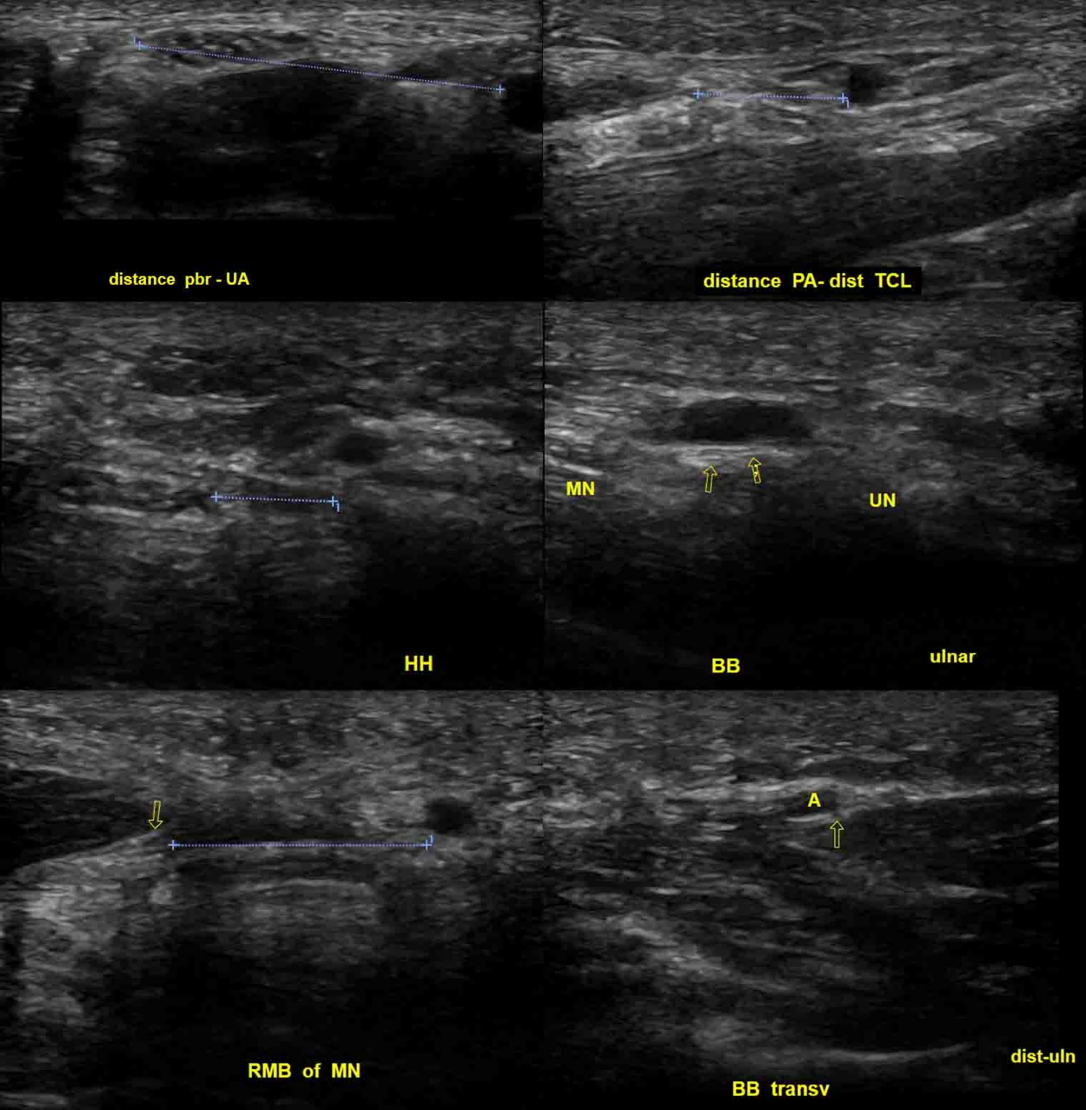
Ultrasound evaluation of the relevant neurovascular anatomy and measurement of important distances according to the sonographic carpal tunnel assessment protocol (Fig. 11). Distance between the PBMN and UA on a transverse image (upper left). Safe zone between the radial end of the ulnar artery and the median nerve at the height of the median nerve on a transverse image. Additionally, the hook of hamate can be visualized and the distance to the median nerve measured (middle left). Distance between the RBMN (arrow) and MN on a transverse image (lower left). Distance between the distal end of the TCR and UA/superficial arterial arch on a longitudinal image (upper right). Visualization of the BB longitudinally (two arrows) on a longitudinally-oblique image just underneath the UA/superficial palmar arch (middle right). Visualization of the BB transversely (arrow) just next to the UA on a longitudinally-oblique image (lower right)

**Fig. 11:**
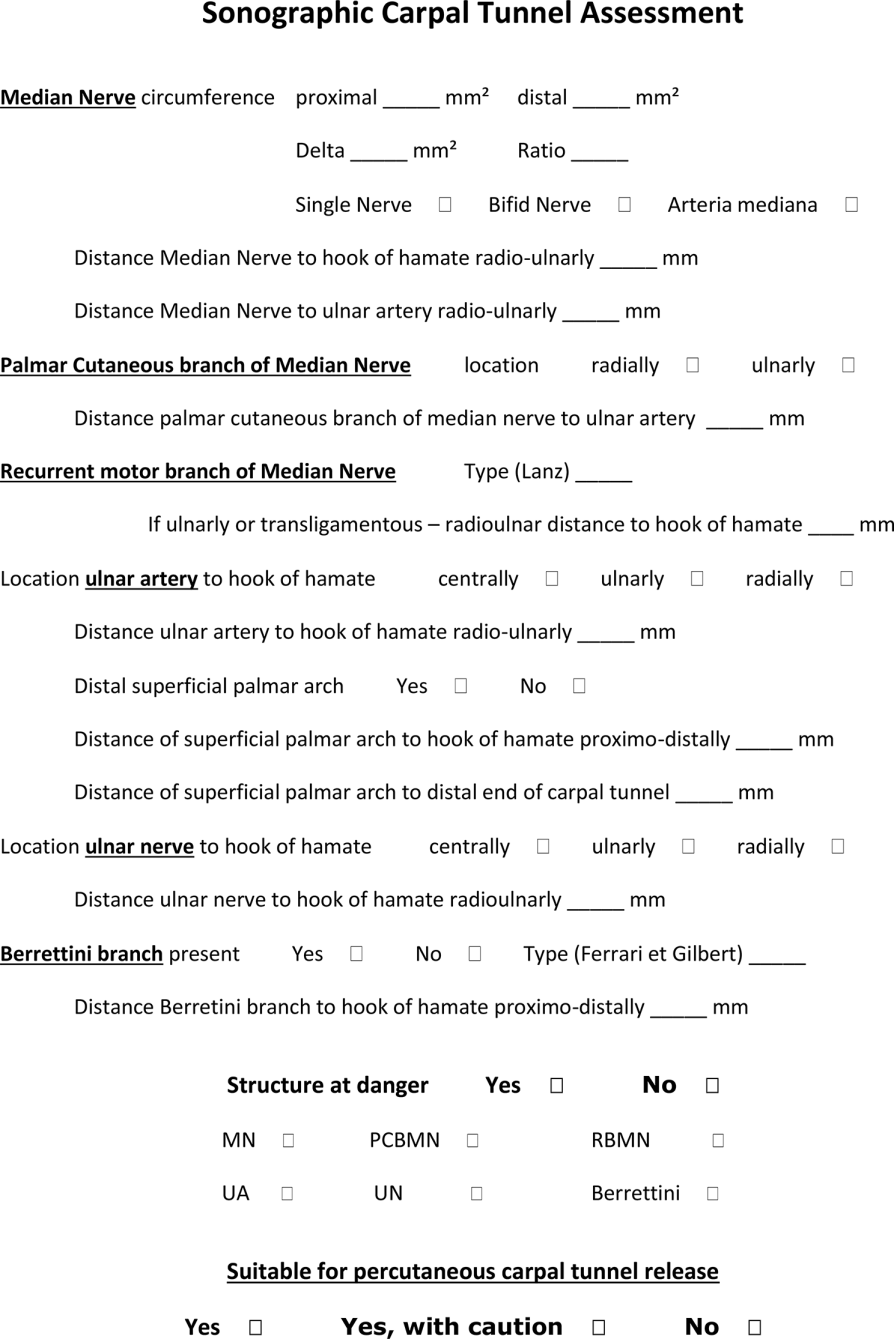
Diagnostic ultrasound evaluation protocol of the carpal tunnel

Ultrasound was able to identify all relevant structures and relevant distances were measurable (Fig. 10) and a diagnostic ultrasound evaluation workflow is proposed in Fig. 11.

**Table 1:** Case counts of distances ≤ 3 mm, < 5 mm and ≤ 5 mm of P1 to neurovascular structures

	≤ 3 mm	< 5 mm	≤ 5 mm
Radioulnar distance MN to P1	16 cases (15%)	32 cases (31%)	52 cases (50%)
Radioulnar distance RBMN to P1	2 cases (2%)	3 cases (4%)	4 cases (4%)
Radioulnar distance LFRF to P1	0 cases (0%)	0 cases (0%)	1 case (1%)
Proximodistal distance BB to P1	4 cases (4%)	11 cases (11%)	15 cases (14%)
Proximodistal distance SPA to P1	5 cases (5%)	16 cases (15%)	18 cases (17%)
Radioulnar distance MN to UA	0 cases (0%)	2 cases (2%)	4 cases (4%)
Radioulnar distance UN to P1	5 cases (5%)	11 cases (11%)	20% (19%)

## Discussion

This study revealed that 26–70% of patients (depending on the safety distance 3–5 mm) exhibit an anatomic configuration of the carpal tunnel that could potentially predispose them to neurovascular injuries of the cutting instrument. The primary structures at risk include the median nerve, the ulnar artery/superficial palmar arch, the Berrettini branch and the recurrent motor branch of the median nerve.

The definition of the safe zone in this study slightly differs from other research. In this study, the transverse safe zone is described as the space between the MN and UA or hook of hamate, depending on which is more radially positioned. This study used the most distal and ulnar point where the hook knife could be inserted as the reference point (P1). The safe zone between P1 and the MN was 6 mm (1–13 mm). Sytsma et al. described a safe zone of 8.5 mm (4.4–12.4 mm) [17]. However, the width of the safe zone can vary depending on the level of measurement (min. 3 mm; max. 17 mm) [16]. The safe zone is larger in men than in women (9.9 mm vs. 7.7 mm) [17], but it does not correlate to the hand size [16]. The width was shown to be smaller in patients with a CTS in comparison to healthy individuals (mean 6.4 mm vs. 4.7 mm), but it can be enlarged significantly by wrist ulnar deviation [18]. Additionally, hydrodissection (which was not conducted in our study) can enlarge the anatomical spaces and distances between all important neurovascular structures and therefore potentially minimize the risk of injury [19].

The division of the third common digital nerve can show different variations. While a distal separation seems to be less prone to injury, a more proximal separation, a bifid nerve and a high origin may increase the risk of injury, as the cutting device can possibly hook the nerve and transect it as shown in Fig. 5 [20; 21]. Ultrasound is feasible to visualize the separation of this branch, and the surgeon can be warned in such cases. Extra caution is essential in such cases (the authors have knowledge of two cases of partial MN injury after an externally conducted ultrasound guided CTR using a hook knife). Patients at high-risk may need to be considered for exclusion from minimal-invasive surgical technique, and an open surgery option could be presented to them.

The RBMN may be at risk especially because of a transligamentous pathway or because of an ulnar origin. Both dangerous situations were also observed and similarly classified clinically during an open CTR [22]. The RBMN was found to originate from the radial aspect or central aspect in 95% of all cases [23]. A dangerous ulnar pathway was present in 2% of patients in our study which is similar to other findings [24]. The risk of transection of this nerve is reported to be 0.5% [25; 26]. The RBMN can adequately be visualized using ultrasound in 100% of all patients using a 18–22 MHz probe and patients with an ulnar or transligamentous pathway can be detected [23].

The PBMN was not in danger in this study due to the ulnar trajectory of the CTR even in transligamentous courses of the PBMN. However, more radial trajectories may harm this nerve, especially in cases of an ulnar take-off of the MN, which can even circle around the palmaris longus tendon on the ulnar side [27]. Cheung et al. reported an ulnar-take off in 4.1% [28]. Our study did encounter such a case only in one case (1%); however, it remained considerably distant from an ulnar-sided trajectory following the long axis of the radial side of the ring finger.

Once more, ultrasound proves highly feasible for reliably visualizing the PBMN [29]. However, caution must be exercised during ultrasound evaluation, as the PBMN might consists of two separate branches [30].

The presence of a BB exhibits significant variability, reported to range from 10 to 100%, with type 3 being the most common [14; 19; 31]. In type 3 configurations, where the BB is positioned distal to the distal end of the TCL, it is less susceptible to injury.

Type 1 and 2 are located near the end of the TCL and therefore prone to iatrogenic injury. Although, Guo et al. state that in the modified thread carpal tunnel release, it is not important to identify a BB because a hydrodissection maneuver can push the median nerve away from the TCL and create a hypoechoic fluid space and the looping path excludes the BB, the authors of the present study have a different opinion [19]. If the nerve is not visualized using ultrasound, it still can be injured using an open technique, endoscopy, a hook knife, or a looping thread technique because it lies directly on the TCL in a Type 1 and 2 in the trajectory of the surgical release as seen in Fig. 8. Therefore, these two types and their distances to the distal end of the TCL need to be searched for meticulously using ultrasound before any surgical intervention. Although, ultrasound is feasible to visualize this nerve branch [32], the authors rather think it is the most difficult part in the preoperative ultrasound carpal tunnel assessment protocol (Fig. 11). Two prerequisites are necessary. First, the ultrasound device needs a high-frequency probe to visualize all small branches. Although, Dukan et al. [32] state that it should be > 10 MHz for visualization of the BB, the authors rather think it should be at least ≥ 18 MHz. The cited study also used a 17 MHz probe. Additionally, a 20–22 MHz probe can visualize all branches more easily than a 18 MHz probe in clinical experience. The second prerequisite is sufficient experience of the operator in nerve ultrasound. The sensitivity and specificity as well as the feasibility of detection of all branches by less experienced operators has not been reported yet.

The UN is situated ulnarly to the UA and usually not in danger during a pathway along the radial side of the longitudinal ring finger axis. The UN was found to be 3.6 mm ulnar to the hook of hamate. However, the range can vary from 5.8 mm radial to 7.5 mm ulnar to the hook. A radial position is however rare (< 10% of patients). Wrist extension leads to an ulnar displacement of the UN and UA [33]. Our study did not encounter such cases and the UN was never in danger.

Yet, the UA was in danger in several cases. Regarding the transverse position, the UA was situated radially to the hook of hamate in 1%, which is less frequent than previously reported (40–56%) [33; 34]. The reason for this discrepancy may be the anatomical dissection and preparation leading to a shifted pathway of the UA. Because of the situation, some authors recommend performing an endoscopic CTR 4–5 mm radial to the hook of hamate, however, this may lead to a higher risk of injuring the MN. An ultrasound technique may possibly ameliorate this situation by visualizing the safe zone adequately [17]. Regarding the longitudinal position of the UA and SPA, the safe zone was 11 mm (0–25 mm) in this study with similar findings in other studies` mean 10–12.7 mm (5–18 mm) [35–37]. There is a high variety regarding the SPA (Fig. 4). There are patients without any arch which are completely safe and there are, rarely, other patients with an arch turning directly at the distal edge of the transverse carpal ligament and therefore highly in danger of injury. (Fig. 7) This longitudinal safe zone depends on the curve of the arch, and the space is bigger more radial than ulnar without significant gender difference [17]. Only 17% of all patients showed a relevant proximal position of the SPA in reference to the ulnar positioned P1. (Table 1) The artery can be injuried by direct dissection because of unawareness. Sometimes the cutting instrument is stuck during an antegrad transection, and the surgeon tries to push harder to transect the TCL. Then with an uncontrolled movement and loss of resistance, the cutting instrument is pushed too far possibly injuring the vessel. Therefore, a retrograde cutting instrument seems rather recommended or a needle technique to avoid this possible complication especially in a close relationship between the distal edge of the TCL and the SPA.

Regarding percutaneous minimal-invasive surgery, endoscopic CTR has the disadvantage that visualization is limited and therefore may lead to a high injury rate of 1.83–1.87% in larger series or even higher rates of up to 9% in smaller series [38]. An ultrasound-guided procedure may potentially be safer because according to existing literature neurovascular structures at risk can be visualized and therefore possibly avoided. To reduce the complication rate, the authors proposed a preoperative ultrasound evaluation of the anatomy of the carpal tunnel according to Fig. 11. Patients at risk with a small safety zone to neurovascular structures should rather be treated either by specialists with sufficient experience and only with special caution using a minimal invasive technique or by open surgery and direct visualization of the carpal tunnel. Similar to our proposition, Nakamichi et Tachibana recommended an open CTR in cases of a small safe zone below 3 mm [16]. There is no definition as to the value of the safety limit. Because hydrodissecton can increase the space the limit of ≤ 3 mm, < 5 mm or ≤ 5 mm may be challenged. Yet, the safe space between the cutting instrument and any endangered structure should be large enough to insert and maneuver the used instrument without touching and tethering any neurovascular structure in proximity.

This study has several limitations. Being an anatomical study, it does not conclusively prove whether a preoperative ultrasound evaluation could reduce the risk of neurovascular injury. Additionally, the anatomical dissections were conducted by students, which might have resulted in accidental trans- or resection of some neurovascular structures, leading to varied frequencies and percentages.

Furthermore, the removal of the skin, subcutaneous tissue, and palmar fascia could have caused slight displacement of neurovascular structures compared to their natural positions. The proximity to a Riché-Cannieu anastomosis (potentially present in 100% of all patients) was not investigated. However, as this branch is a connection between the RBMN and the deep UN lying far radially, it does not seem to be in danger of injury while using an ulnar trajectory for CTR. Because the skin and subcutaneous tissue was already removed, precise investigation of any subcutaneous nerve connections between the median and ulnar nerve at the incision area could not be studied. Lastly, the study did not investigate any ultrasound findings, therefore the study cannot prove if any identified structure by ultrasound is really the mentioned structure. This would have been needed to be confirmed openly afterwards which was not possible. However, previous work as referenced in the discussion section already showed a high specifity of ultrasound regarding the neurovascular structures.

## Conclusion

In conclusion, certain patients may inherently face an elevated risk of neurovascular injuries during minimally invasive carpal tunnel releases due to their anatomical variations. Four potentially risky scenarios were clearly illustrated. Consequently, one may consider conducting a preoperative ultrasound assessment of neurovascular structures at risk, when endoscopic or ultrasound-guided tunnel release are planned. In high-risk patients, open surgery should be preferred.

## Data Availability

Not applicable for this article.
